# Genomic-Wide Analysis with Microarrays in Human Oncology

**DOI:** 10.3390/microarrays4040454

**Published:** 2015-10-16

**Authors:** Kenichi Inaoka, Yoshikuni Inokawa, Shuji Nomoto

**Affiliations:** Department of Surgery, Aichi-Gakuin University School of Dentistry, 2-11 Suemori-dori, Chikusa-ku, Nagoya 464-8651, Japan; E-Mails: inaokakn@dpc.agu.ac.jp (K.I.); yinokawa@med.nagoya-u.ac.jp (Y.I.)

**Keywords:** microarray, gene expression, combination array

## Abstract

DNA microarray technologies have advanced rapidly and had a profound impact on examining gene expression on a genomic scale in research. This review discusses the history and development of microarray and DNA chip devices, and specific microarrays are described along with their methods and applications. In particular, microarrays have detected many novel cancer-related genes by comparing cancer tissues and non-cancerous tissues in oncological research. Recently, new methods have been in development, such as the double-combination array and triple-combination array, which allow more effective analysis of gene expression and epigenetic changes. Analysis of gene expression alterations in precancerous regions compared with normal regions and array analysis in drug-resistance cancer tissues are also successfully performed. Compared with next-generation sequencing, a similar method of genome analysis, several important differences distinguish these techniques and their applications. Development of novel microarray technologies is expected to contribute to further cancer research.

## 1. Introduction

Microarray technology has been widely used for various fields such as medical science and basic biology. These methods allow the analysis of exhaustive gene expression changes in specimens which were carried out for their genome analysis. Over the last decade, the number of studies using DNA microarray has rapidly increased, and a PubMed search for “DNA microarray” reveals more than 80,000 publications in May 2015. In particular, many studies using this technology are in the field of oncology, and these studies have identified a number of critical genes in cancer progression. In this review, we provide an overview of the development of principle microarrays and the application of each array, and we introduce initial findings using the combination array. The last section describes the application, limitations, association with next-generation sequencing, and future prospects.

## 2. The Initial History of Microarrays

The original DNA array was created with the colony hybridization method of Grunstein and Hogness [1]. In this method, the DNA of interest is cloned into *Escherichia coli* plasmids, and *E. coli* colonies with different hybrid plasmids can be screened to determine a specified DNA sequence or gene. DNA prints of the colonies are then hybridized to radioactive RNA, and are analyzed by autoradiography. This method can be used to isolate any gene.

Using this approach, Gergen *et al.* [2] reported a method for making paper filter replicas of such an ordered collection and developed a strategy for creating a high-density (10,000 colonies/petri) unordered collection. These different mixtures of probes could be used for nucleic acid hybridization screens of recombinant DNA colonies.

In 1980, Crampton *et al.* [3] compared RNA populations derived from normal human lymphocytes and fibroblasts by hybridizing each RNA to cDNA derived from the other RNA population. The isolation of cloned cDNA sequences revealed the differentially expression between two samples.

Schena *et al.* [4] published a high-capacity system that was developed to analyze the gene expression in parallel. Microarray technologies which were prepared by high-speed robotic printing of complementary DNAs on glass were useful for quantitative expression analysis of the corresponding genes. Differential gene expression measurements were obtained using simultaneous, two-color fluorescence hybridization.

In 1996, DeRisi *et al.* [5] published a method describing very high density cDNA microarrays on glass substrates using fluorescent probes, and these arrays were used to search for differences in gene expression associated with tumor suppression.

Since these initial studies, DNA microarray technologies have developed rapidly in a variety of fields.

### 2.1. Microarray Devices (DNA Chip Synthesis)

#### 2.1.1. *In Situ* Synthesized Type

The methods for preparing DNA chips combine photochemistry and photolithography with solid-phase DNA synthesis chemistry to generate a high-density oligonucleotide probe array *in situ* [6,7,8]. These two-dimensional arrays containing hundreds or thousands of oligonucleotide probes provide a powerful DNA sequence analysis tool [6]. For example, this method is now used to produce the high-density gene chip probe arrays, which are used for the detection and analysis of point mutations and SNPs and for gene expression studies [9]. The change of the carried probe number in expression array by Affymetrix shows Table 1.

**Table 1:** The change Probe/GeneChip number in expression arrays by Affymetrix.

Date	Feature Size	Probe/GeneChip
1994	100	16,000
1996	50	65,000
1998	24	256,000
2000	20	400,000
2002	18	505,000
2003	11	1,354,000
2004	8	2,560,000
2005	5	6,553,000

#### 2.1.2. Spotting Type

Many microarray spotting technologies and techniques have been successfully developed. DNA chips can be produced using the spot method, in which cDNAs are selectively deposited on specific positions on a glass slide using a spotter. Two of the more important spotting techniques used are the pin-based fluid transfer systems [10,11,12,13] and the piezo-based inkjet dispenser systems [14]. Table 2 shows the change of the coverage of genes and transcripts in expression array by Agilent. The microarray devices have rapidly developed.

**Table 2:** The change of the coverage of the genes and transcripts in expression array by Agilent.

Date	Gene Transcript
2000	12,814
2002	15,217
2006	20,356
2010	41,000
2014	56,689

## 3. Microarray Types

### 3.1. Expression Array

The major application of DNA microarrays has been for the measurement of gene expression levels. RNA is extracted from cells, directly fluorescently labeled, and converted to labeled cDNA. The labeled cDNA is hybridized to the microarray, the array is washed, and the signal is detected by measuring fluorescence at each spot. The intensity of the signal on each spot is taken as a measure of the expression level of the corresponding gene [9,15].

Multiple studies have successfully used these techniques to evaluate gene expression levels in human diseases, including cancers. Shim *et al.* [16] performed an expression profile of genes associated with human cervical cancer using cDNA expression arrays. Rhee *et al.* [17] showed molecular evidence of the qualitative and quantitative high heterogeneity in gene expression among three human glioblastoma cell lines using cDNA expression arrays.

### 3.2. Methylation Array

Cancers often exhibit aberrant methylation status of gene promoter regions associated with loss of gene function [18,19]. This epigenetic process acts as an alternative strategy to mutations to disrupt tumor suppressor gene function. CpG island hypermethylation has been shown to be a common event in cancers [20,21]. To detect hypermethylation status, the demethylating agent 5-Aza-2′-deoxycytidine is used [22] and gene expression changes are subsequently measured by microarrays [23]. Methylation arrays can also aid in the identification of three tumor suppressor genes including *CRIP-1*, *Apolipoprotein D*, and *Neuromedin U* by comparing the methylation of CpG islands of promoter regions in cancer tissue and corresponding normal tissue [24]. Notably, the demethylation status of particular genes is also related to carcinogenesis. Genes upregulated by demethylation can play a clinically significant role in cancer tissues [25].

### 3.3. Comparative Genomic Hybridization (CGH) Array

The CGH method measures genomic changes such as deletions of chromosome copy number and amplification [26]. The CGH array was developed as a method to detect genome abnormalities such as the minute gene amplification, deletion, and DNA copy number alterations [27,28,29,30,31,32,33].

### 3.4. Single Nucleotide Polymorphism (SNP) Array

A SNP, a variation at a single site in DNA, is the most frequent type of variation in the genome [34,35], with an estimated 10 million SNPs in the human genome [36]. SNPs have been associated with disease and drug metabolism. The SNP array is a type of DNA microarray used to detect polymorphisms within a population [37,38,39,40] and this array can detect SNPs associated with diseases [41,42], genotyping [40,43,44,45], copy number variation [46], and loss of heterozygosity (LOH) [38,39].

### 3.5. MicroRNA(miRNA) Array

In 1993, Lee *et al.* [47] discovered that lin-4, a gene known to control the timing of *Caenorhabditis elegans* larval development, does not code for a protein but instead produces a pair of small RNAs approximately 22 and 61 nt in length. The shorter lin-4RNA is now recognized as the founding member of an abundant class of short regulatory RNAs called microRNAs or miRNAs [48,49,50]. The importance and the role of miRNA-directed gene regulation are coming into focus as their regulatory targets and functions [51]. Liu *et al.* [52] described the using of the first miRNA microarray. After that, the miRNA microarrays revealed their functions in control of cell proliferation, cell death associated with carcinogenesis [53,54], fat metabolism in flies [55,56], and modulation of hematopoietic lineage differentiation in mammals [57]. For example, miR-21 was detected by miRNA array in various cancers [58], and high miR-21 expression is associated with the poor survival and poor therapeutic outcome in colon cancer [59].

### 3.6. Long-Noncoding RNA (LncRNA) Array

More recently, lncRNAs, generally defined as short RNAs greater than 200 nt in length, have risen to prominence with important roles in a broad range of biological processes [60]. LncRNAs regulate gene expression at the level of post-transcriptional processing such as protein synthesis, RNA maturation, transport, cell differentiation, immune responses, and activity and localization of protein coding genes [61,62]. They also exert their effects in transcriptional gene silencing through the regulation of chromatin structure [60,63]. Dysregulation of lncRNAs is associated with many human diseases, including various types of cancers [64]. Many studies have used lncRNA microarrays to demonstrate lncRNA gene expression profiles and the prognostic potential of lncRNA profiles in various cancers [65,66].

### 3.7. Platform Description

The presently available and most used platforms show Table 3. These platforms are used widely in many laboratories.

**Table 3:** List of the main platforms ion the each microarrays.

Vendor	Expression Array	Methylation Array	SNP Array	miRNA Array
Affymerix	Human GenomeU133 Plus 2.0 Array	-	Genome-wide human SNP Array 6.0	GeneChip^®^ miRNA 4.0 Array
Illumina	HumanHT-12 v4 Expression BeadChip Array	Human Menthylation450 BeadChip	HumanOmniExpress BeadChip Kit	-
Agilent	SurePrint G3 Human Gene Expression v3 8 × 60 K Microarray Kit	Human DNA Methylation Microarray 244 K	SurePrint G3 CGH + SNP Microarray Kit, 2 × 400 K	SurePrint G3 Human miRNA Microarray 8 × 60 K

## 4. Applications of Microarrays

### 4.1. Comparison with Cancer Tissue and Corresponding Normal Tissue

Many studies have used microarrays to compare cancer regions with non-cancer regions and detected many cancer-related genes such as oncogenes and tumor suppressor genes [67,68,69]. For example, expression arrays have been successfully used to examine esophageal cancer [70,71], gastric cancer [72,73], colorectal cancer [67], breast cancer [74], and HCC [75,76,77,78], and methylation arrays were used in esophageal cancer [79], gastric cancer [80,81,82], colorectal cancer [83], breast cancer [21,84], and HCC [85,86,87].

### 4.2. Combination Array

Recently, the new microarray methodology have reported more effective. Nomoto *et al.* [88] developed the “double-combination array” by combining expression array analysis and SNP array analysis to effectively gain whole genome information. The gene expression profile provides a snapshot of the transcriptional state of noncancerous and tumor tissues. The SNP array is a useful tool for surveying LOH, a prominent characteristic of many human cancers. The authors combined the use of these two microarrays in one representative surgical sample and effectively identified several, novel tumor-specific gene alterations [89].

First, in one cancer sample, novel target genes that are downregulated in cancer regions are detected by expression array. The silencing mechanism is then analyzed using the SNP array, and the results confirm the absence of copy number variation and LOH. Assuming that the observed downregulation may be due to epigenetic changes, the target genes are validated in clinical specimens using methylation-specific polymerase chain reaction. Positive results can suggest that the gene is downregulated in cancer tissues through promoter hypermethylation and may have a role as a candidate tumor suppressor gene. This technique has detected many novel candidate tumor suppressor genes.

Using this double-combination array, many important genes have been detected, including metallothionein 1G (*MT1G*), epidermal growth factor-containing fibulin-like extracellular matrix protein 1 (*EFEMP1*), A kinase anchor protein 12 (*AKAP12*), and leukemia inhibitory factor receptor (*LIFR*) genes as tumor suppressor genes in hepatocellular carcinoma, and reelin (*RELN*) as a key regulatory gene associated with the recurrence of HCC [88,89,90,91,92]. Likewise, Kobayashi *et al.* [93] detected the suppressor of cytokine signaling 4 (*SOCS4*) as a novel gastric cancer suppressor gene using a double-combination array in gastric cancer.

To further evaluate hypermethylation of the promoter CpG islands, methylation array can be added to complete the triple-combination array method, which is designed to more efficiently search for epigenetic alterations [94,95]. The triple-combination array has detected many genes, including bleomycin hydrolase gene (*BLMH*), estrogen receptor 1 gene (*ESR1*), dynamin 3 (*DNM3*), doublecortin domain-containing 2 (*DCDC2*), collagen type 1 alpha 1 gene (*COL1A1*), protein tyrosine kinase 7 (*PTK7*), and cyclin J (*CCNJ*) as candidate cancer-related genes in HCC [94,95,96,97,98,99,100].

### 4.3. Combination Array in other Groups

Other studies have successfully used the combination of expression array and SNP array in examining infiltrating ductal carcinoma of the breast [101], the combination of methylation array and expression array in prostate cancer [102], and the combination of expression array and CGH array in renal cell carcinoma [103].

### 4.4. Selection of Comparison Samples

In general, microarrays are typically used to compare tumor tissue and corresponding normal tissue. However, when used to compare precancerous tissue with the corresponding normal tissue, it can identify alterations in gene expression and methylation events that lead to carcinogenesis, and thus may have the potential to evaluate the risk of carcinogenesis and recurrence.

Nomoto *et al.* [104] examined adjacent nonneoplastic liver tissue from a patient with hepatocellular carcinoma comparing with supernormal liver (SN) samples taken from metastatic secondary malignancies of the liver. The tissue of SN was actual normal liver. Therefore, it seemed that there was no molecule which showed fundamentally abnormal. However, it could not be denied that there was the individual feature. Then they thought that this individual projection was erased by mixing 11 SN samples. Expression profiling and methylation arrays revealed that expression of the thimet oligopeptidase (*THOP1*) gene in the background liver of HCC is likely to be a good biomarker for risk of HCC development.

To validate genome-wide DNA methylation profiles during multistage hepatocarcinogenesis, Ammerpohl *et al.* [105] revealed that the methylation status have changed gradually from normal to cirrhosis and further to HCC using a methylation array. Nagashiro *et al.* [106] established criteria for carcinogenetic risk estimation based on DNA methylation array profiling to compare samples of noncancerous liver tissue obtained from HCC patients with normal liver tissue samples. Arai *et al.* [85] performed bacterial artificial chromosome (BAC) array-based methylated CpG island amplification using a microarray of 4361 BAC clones in normal liver tissue obtained from patients without HCC, noncancerous liver tissue obtained from patients with HCC, and HCC samples. The DNA methylation status of the 41 BAC clones was correlated with the cancer-free and overall survival rates of patients with HCC.

Okamoto *et al.* [107] classified patients with hepatitis C-positive HCC into two groups: the single nodular HCC group and multicentric (MC) HCC group. The authors compared gene expression patterns of the noncancerous liver tissue specimens using cDNA microarrays, and created a scoring system to estimate the risk for MC hepatocarcinogenesis. Utsunomiya *et al.* [108] performed miRNA microarrays to compare the miRNA expression patterns in the non-cancerous liver tissues between the MC recurrence group and no MC recurrence group to identify miRNAs related to MC recurrence. The authors detected 20 differently expressed miRNAs, 18 of which were downregulated in the MC group and 2 of which were upregulated.

Sato *et al.* [109] performed genome-wide DNA methylation array analysis in normal lung tissue obtained from patients without any primary lung tumor, non-cancerous lung tissue obtained from patients with lung adenocarcinomas, and tumorous tissue. DNA hypermethylation at precancerous tissues was strengthened during progression to lung adenocarcinomas.

### 4.5. Analysis of Drug-Resistant Cancer Tissues

Microarray technology has been successfully used to define the molecular changes associated with the drug-resistant phenotype in drug-resistant cancer cells [110]. These changes may be useful as biomarkers of drug sensitivity, molecular target medicines and prediction factors of chemotherapy response.

Duan *et al.* [110] showed that paclitaxel- and adriamycin-resistant ovarian cancer cell lines had significant overexpression of at least one cytokine/chemokine compared with their drug-sensitive parent line. Liu *et al.* [111] detected that maternally expressed gene 3 (*MEG3*) expression was markedly decreased in cisplatin-resistant A549/DDP cells compared with parental A549 cells as shown by an lncRNA microarray. Patients with lower levels of MEG3 expression also showed worse responses to cisplatin-based chemotherapy. Thus, MEG3 may represent a new marker of poor response to cisplatin and could be a potential therapeutic target for lung cell adenocarcinoma chemotherapy. Gao *et al.* [112] showed that cluster of differentiation 44 (*CD44*) was overexpressed in drug-resistant ovarian cancer cell lines, and the authors performed a unique ovarian cancer tissue microarray constructed with paired primary, metastatic, and recurrent tumor tissues from individual patients. Both the metastatic and recurrent ovarian cancer tissues expressed higher levels of *CD44* than the primary tumor. Additionally, *CD44* knockdown in ovarian cancer cells increased sensitivity to the anticancer drug paclitaxel. Thus, these findings demonstrated that upregulation of *CD44* was a crucial event in the development of the recurrence, metastasis, and acquisition of drug resistance in ovarian cancer. Fang *et al.* [113] studied miRNA expression profiles in colorectal cancer, comparing chemoresistant and chemosensitive groups by microarray analysis. Overexpression of miRNA-17-5p was found in chemoresistant patients. The authors also found that *PTEN* was a target of miR-17-5p in colon cancer cells, and their context-specific interactions were responsible for multiple drug resistance. Akcakaya *et al.* [114] performed a miRNA array in drug-resistant gastrointestinal stromal tumors, and Maeda *et al.* [115] validated the gene alterations in drug-resistant gastric cancer by expression and methylation arrays.

## 5. The Present and Future of Microarrays

### 5.1. Effective use of Public Databases for Microarray Data

Microarray expression studies are producing massive quantities of gene expression and other functional genomic data, which will help provide key insights into gene function. It is widely acknowledged that there is a need for public repositories for microarray data [116] whose functions would include providing free access to supporting data for publications based on microarray experiments. Such repositories are under development by the National Center for Biotechnology Information (which has developed the Gene Expression Omnibus) [117], the DNA Database of Japan [118], and the European Bioinformatics Institute (which has developed ArrayExpress) [119]. The miRBase database is also a searchable database of published miRNA sequences and annotation [120].

In addition, the system can search for a specific gene and a specific disease and use properly for each purpose. It is necessary for other researchers to be careful to have access to the underlying data. Even the most carefully conducted studies should require intensive review and consideration of previously published data before embarking on new studies [121].

Although many microarray results have been derived from public databases, one problem was the lack of standards for presenting and exchanging such databases. To address these issues, the members of the Function Genomics Data Society created the MIAME (Minimum Information About a Microarray Experiment) standards for the description of microarray experiments [122]. Making microarray data public in a MIAME-compliant manner has become a precondition for publication for many journals [117]. Publishing original data and protocols facilitates independent evaluation of results and re-analysis, and maintains the spirit of open access [123].

### 5.2. The Relevance of Microarray Quality Control

DNA microarray technologies have had some problems regarding reproducibility and comparability between laboratories and across inter- and intra-platforms of gene expression measurements [124,125,126,127,128,129,130]. The MicroArray Quality Control (MAQC) project was initiated to address these concerns and showed intra-platform consistency across test sites as well as a high level of inter-platform concordance in terms of genes identified as differentially expressed by microarray methods. This study provides a resource that represents an important first step toward establishing a framework for the use of microarrays in clinical and regulatory settings [131,132]. International organizations such as External RNA Control Consortium [133], the Microarray Gene Expression Data Society [123], and the MAQC project are providing the microarray community with standardization of data reporting, common analysis tools, and useful controls that can help provide confidence in the consistency and reliability of these gene expression platforms [131].

### 5.3. Next-Generation Sequencing (NGS) Compared with Microarrays

Recently, the advent of NGS, or massively parallel sequencing, has precipitated the discovery of variants in the human genome [134], allowed whole-genome sequencing of microorganisms [135], and has led the way towards novel applications in the fields of human genetics [136], cancer [137,138], and infectious diseases [139,140]. NGS technologies have had a great impact on the field of expression research. Compared to microarray technology, the NGS method has several distinct advantages. The detection range of NGS is not limited to a set of predetermined probes as with the microarray technology, therefore NGS is capable of identifying new genes. And, the analysis of a microarray is limited to the gene level for most arrays, whereas NGS can detect expression at the gene, transcript, and coding DNA sequence levels. Finally, NGS can be used for traditional transcriptome profiling [141,142], identification of novel transcripts [143], identification of expressed SNPs [144,145], alternative splicing, and for the detection of gene fusion events [146,147,148,149]. However, in comparison with a microarray, NGS provides enormous gene information and thus requires significant costs for analysis [132,150,151]. Therefore, it will be necessary to use each characteristic effectively.

## 6. Conclusions

Development of microarray technologies and their applications have been rapidly advancing, and significant amounts of raw data have already been generated. Using these data effectively will enable researchers to further studies in the areas of understanding human disease, with the aim of improving diagnosis and developing effective treatments for many diseases, including cancer.
